# Color Stability of a New Rice Husk Composite in Comparison with Conventional Composites after Exposure to Commonly Consumed Beverages in Malaysia

**DOI:** 10.1155/2019/9753431

**Published:** 2019-05-02

**Authors:** Kacharaju Kranthi Raja, Padmini Hari, Melissa Queen Kha Chin, Kiran Singbal, Ismail M. Fareez

**Affiliations:** ^1^Department of Conservative Dentistry, Faculty of Dentistry, MAHSA University, Bandar Saujana Putra, 42610 Jenjarum, Selangor Darul Ehsan, Malaysia; ^2^Faculty of Dentistry, MAHSA University, Bandar Saujana Putra, 42610 Jenjarum, Selangor Darul Ehsan, Malaysia; ^3^Department of Periodontology, Faculty of Dentistry, MAHSA University, Bandar Saujana Putra, 42610 Jenjarum, Selangor Darul Ehsan, Malaysia; ^4^Department of Oral Biology and Biomedical Sciences, Faculty of Dentistry, MAHSA University, Bandar Saujana Putra, 42610 Jenjarum, Selangor Darul Ehsan, Malaysia

## Abstract

**Objective:**

To evaluate the color stability of a new organic rice husk nanocomposite as compared to four conventional composites after exposure to commonly consumed beverages in Malaysia.

**Methods:**

One hundred and twenty-five disk samples were prepared from a new rice husk-based composite and four other conventional methacrylate-based light-cured composites of shade A2. The samples were immersed in four commonly consumed beverages: coco-based drink, kopi, Chinese tea, and teh tarik for four weeks. The color measurements were carried out every week using the reflectance spectrophotometer according to the CIE *L*^*∗*^*a*^*∗*^*b*^*∗*^ color system. Color changes of samples (Δ*E*) in each week were calculated. Statistical analysis was carried out by performing a mixed ANOVA and Tukey's post hoc test in order to analyse the differences in Δ*E*.

**Results:**

The findings revealed a statistically significant difference of Δ*E* reading (*p* < 0.05) among all composites immersed in all four beverages after four weeks. Rice husk composites exhibited lesser color stability as compared to Ceram.X One Universal (*p* < 0.001) and G-aenial Universal Flo (*p* < 0.001) but showed higher color stability compared to Solare-X (*p* < 0.001) and Neofil (*p* < 0.001). Coffee and Chinese tea had the most significant impact on color changes (*p* < 0.05) observed in all composites over four weeks of study.

**Conclusion:**

Rice husk composite showed acceptable color stability. It can be considered as an alternative to conventional composites due to its eco-friendly properties.

## 1. Introduction

The escalating emphasis on aesthetics has resulted in great improvements in dental composites, offering excellent optical properties to meet the demands of the patients [1]. A good composite restoration should simulate the appearance of a natural tooth [2, 3]. Color stability of a material plays an important role in deciding the success or failure of a composite [4]. Aesthetic failure of restorations caused by staining or discoloration has resulted in many replacements [5]. Generally, degrees of extrinsic and surface discoloration in the composites vary according to the oral hygiene and dietary and smoking habits. Subsurface and intrinsic discolorations restoration is closely related to the composition that is affected by factors such as (1) type of the resin matrix which influences the hydrophilic or hydrophobic nature of the material and thus its water absorption ability, (2) filler particle size and distribution, and (3) the degree of polymerization [5–7].

There is only limited literature available on the color stability of the commercially available four methacrylate-based composites, namely, G-aenial Universal Flo, Solare-X, Ceram.X One Universal, Neofil. The present study investigated the color stability of these materials and a novel rice husk composite [8], which is an organic nanocomposite that contains amorphous rice husk nanosilica. The nanosilica obtained from rice husk was silanized with 6 wt.% MPS. The treated fillers were dried in a hot air oven at 80°C for 24 h. 0.01 wt.% camphorquinone and 0.01 wt.% DMAEMA used as the photoinitiator system were dissolved in the matrix resin (Bis-GMA/TEGDMA, 60/40 wt.%.) under subambient light. The silanized fillers were then incorporated into the matrix phase and thoroughly mixed to obtain a homogenous paste. A ratio of 50 : 50 filler/matrix was used in the current study to prepare the dental nanocomposite [8]. The flexural strength, flexural modulus, compressive strength, hardness, and surface roughness tests of this novel rice husk composite though inferior were found to be comparable a commercially available composite (Z 250) [9].

Dietary habits of the Malaysian populace indicate a substantial daily intake of beverages such as coco-based drink (Milo), kopi (coffee), Chinese tea, and teh tarik (milk tea). Thus, their effect on the color stability of composite restorative materials is highly relevant and worth investigation. It has been found that the staining effect of a beverage on the composite is related to the amount and frequency of its intake [3]. Nevertheless, to date, only few studies reported the staining effect of coco-based drink, milk tea, and pu-erh tea.

Spectrophotometry has been reported as the reliable technique to perform quantitative color assessment in dental material studies. To assess chromatic differences, the American Dental Association has recommended the use of Standard Commission Internationale de I'Eclairage (CIE *L*^*∗*^*a*^*∗*^*b*^*∗*^) color differential system which is well suited for determination of minor color differences and has been widely used in previous studies [2, 6].

Therefore, the aim of the present study was to evaluate the color stability of rice husk-based composite and four other commercially available methacrylate-based composites after immersion in the commonly used beverages. The null hypothesis tested is as follows: (1) rice husk-based composite would not elicit better color stability compared to conventional methacrylate-based composite and (2) the types of beverages used and exposure time of immersion study would not influence the color of the rice husk composite.

## 2. Materials and Methods

**Figure 1:**
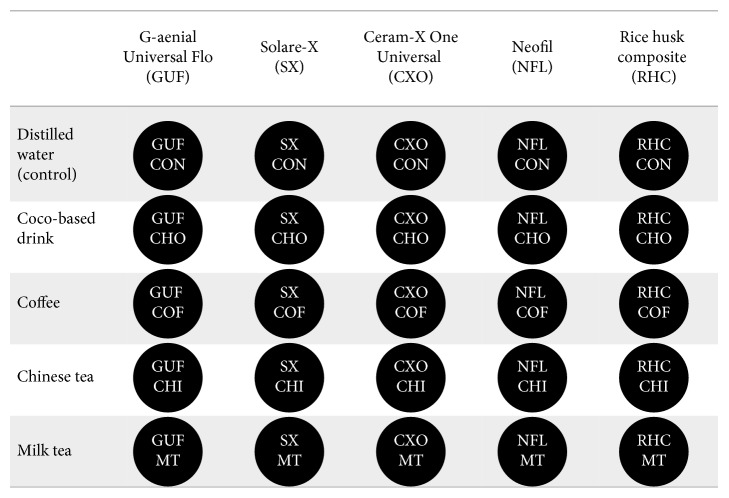
The disc samples used to evaluate the color stability of rice husk-based composite and four other commercially available methacrylate-based composites (*n*=5).

Five methacrylate-based composites including rice husk-based composites were evaluated in this study (Table 1). One hundred and twenty-five disk samples were fabricated from five different resin-based composite materials of shade A2 by condensing the material into a standardized metal mould with a diameter of 10.0 mm and height of 2.0 mm. The mould with the uncured composite resin covered with the mylar strip was pressed between two glass plates to remove the excess material and obtain a flat and smooth surface. All samples were polymerized by the LED light cure unit (LUX E, Guilin Woodpecker, Guangxi, China) using 1,200 mW cm^−2^ of irradiance and standard polymerization mode for 40 s of exposure on both the surfaces. Specimens were numbered on the bottom by using a high-speed small round bur and stored in distilled water at room temperature for 24 hours for rehydration and completion of the polymerization. The discs were labeled as indicated in Figure 1. All specimens were not subjected to any polishing procedure to minimize the effect of polishing on stain intake by the various composite materials as we wanted to test the effect of composition on the staining ability.

### 2.1. Beverages

As shown in Table 2, the beverages used in the study are as follows: coco-based drink (15.0% w/v, Milo, Nestlé, Selangor, Malaysia), coffee (3.0% w/v, NESCAFE®, Nestlé, Selangor, Malaysia), Chinese tea (4.0% w/v, TaHongYin Puer, Yunnan, China), and milk tea, as prepared by mixing black tea (6.0% w/v, Boh, Selangor, Malaysia) and sweetened condensed milk (20% w/v, Fraser & Neave, Selangor, Malaysia).

### 2.2. Color Measurements

Before the immersion experiment commenced, the baseline color measurements were conducted according to the CIE *L*^*∗*^*a*^*∗*^*b*^*∗*^ (Commission Internationale de I'Eclairage, *L*^*∗*^, *a*^*∗*^, *b*^*∗*^) system. Twenty-five disc specimens were prepared for each composite to test in 5 different staining solutions that correspond to a total of 125 samples. The specimens were rinsed with distilled water and wiped dry with gauze before being immersed in staining solutions. Baseline color measurements (*T*_0_) were made. Every subgroup of five composite samples was immersed in beverages at room temperature for four weeks. Color measurements were then made at a time interval of 1 week (*T*_1_), 2 weeks (*T*_2_), 3 weeks (*T*_3_), and 4 weeks (*T*_4_). Beverages were covered to prevent evaporation and replaced every 2 days to avoid bacteria or yeast contamination [2, 10]. All the measurements were performed using a reflectance spectrophotometer (Spectrophotometer CM-5, Konica Minolta, Osaka, Japan) with a wavelength of 360 to 740 nm. For each specimen, an average of three measurements was taken. Using the CIE *L*^*∗*^*a*^*∗*^*b*^*∗*^ system, differences in color (∆*E*) and color coordinates (Δ*L*^*∗*^, Δ*a*^*∗*^, and Δ*b*^*∗*^) between baseline (*T*_0_) and *T*_1_, *T*_2_, *T*_3_, and *T*_4_ measurements were calculated by using the following formula [2, 5]:

(1) $$\begin{array}{c} \Delta E=\sqrt{{\left(L_{2}-L_{1}\right)}^{2}+{\left(a_{2}-a_{1}\right)}^{2}+{\left(b_{2}-b_{1}\right)}^{2}}. \end{array}$$

### 2.3. Statistical Analysis

Statistical analysis was performed by using Mauchly's test (corrected by using Greenhouse–Geisser estimates of sphericity) and Tukey's post hoc test in order to evaluate differences of ∆*E* between groups. The data analysis was carried out using statistical software (SPSS for Windows, version 22.0; SPSS Inc., Chicago, UIL, USA), with the significance level of *p* < 0.05. A three-way ANOVA test was performed for the intragroup comparison, and the difference in ∆*E* is statistically significant in the comparison between staining agents in each composite group, and vice versa, except for the control group.

## 3. Results

**Figure 2:**
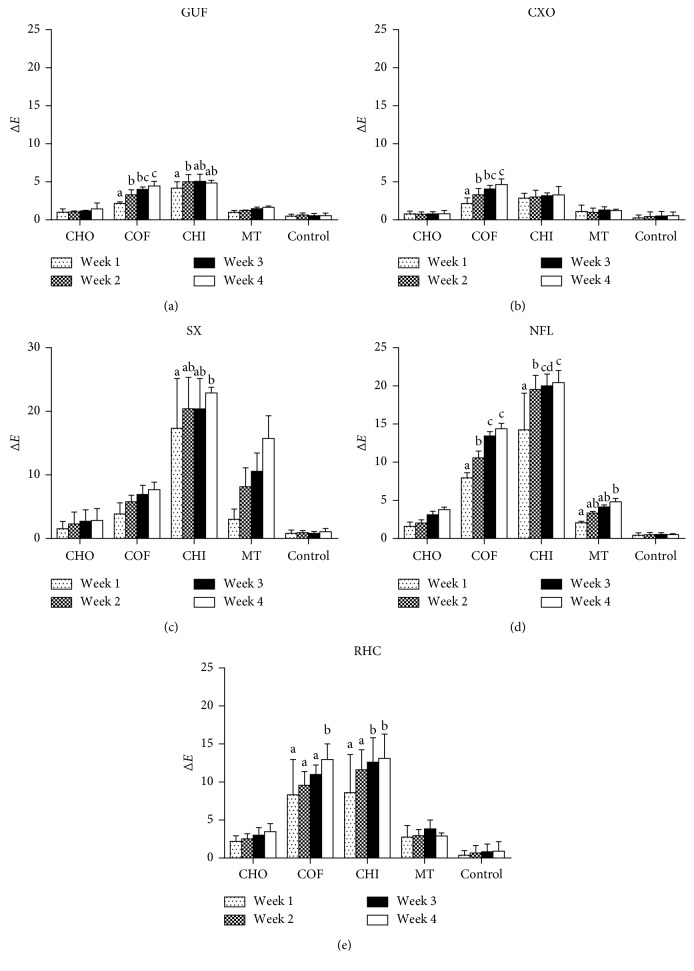
Color changes of composites across four weeks of the immersion period. G-aenial Universal Flo, GUF; Solare-X, SX; Ceram.X One Universal, CXO; Neofil, NFL; rice husk composite, RHC.

The mean color change (∆*E*) values of composites after four weeks of immersion in five different staining solutions are presented in Figure 2 and summarized in Table 3. The ∆*E* values higher than 2.7 (with 50 : 50% confidence level) will be considered as clinically unacceptable staining [11]. Post hoc test results revealed statistically significant difference (*p* < 0.05) in the color change of the test composites, following 4 weeks of immersion in the five different staining solutions except that ∆*E* found between CXO and GUF and between NFL and SX (*p* > 0.05). CXO and GUF showed unacceptable ∆*E* after immersion in coffee (∆*E* = 4.65 and 4.44, respectively) and Chinese tea (∆*E* = 3.29 and 4.85, respectively), after four weeks of study. RHC and NFL exhibited unacceptable ∆*E* in all the staining solutions at the end of four weeks. Overall, the color stability of the composites was ranked in the following order: CXO ≥ GUF > RHC > NFL ≥ SX, with significant difference observed among CXO > RHC (*p* < 0.001), CXO > NFL (*p* < 0.001), CXO > SX (*p* < 0.001), GUF > RHC (*p* < 0.001), GUF > NFL (*p* < 0.001), GUF > SX (*p* < 0.001), RHC > NFL (*p* < 0.001), and RHC > SX (*p* < 0.001). SX showed significantly high ∆*E* in Chinese tea (∆*E* = 22.89) and milk tea (∆*E* = 15.73).

All of the tested beverages have resulted in unacceptable ∆*E* in all the composites, except the coco-based drink, Milo. The staining potential of beverages was ranked in the following order: Chinese tea > coffee > milk tea > coco-based drink with a significant difference observed among all beverages (*p* < 0.001). Coffee has stained all the composites beyond the clinically acceptable level, but NFL and RHC are being stained more significantly than the other counterparts. Among the composites tested, the highest discoloration was observed in the SX composite after immersion in Chinese tea (∆*E* = 22.89) at 4 weeks. The staining effect of milk tea on SX was also surprisingly profound (∆*E* = 15.73) as compared to other composites. Coco-based Milo drink has stained all the composites to clinically unacceptable level, except GUF (∆*E* = 1.45) and CXO (∆*E* = 0.80), which manifested ∆*E* close to that of control (∆*E* = 0.50). Only SX manifested clinically perceptible ∆*E* (∆*E* = 1.06) in distilled water, whereas the other composites showed imperceptible ∆*E*. Control group showed the lowest staining potential overall. There is no significant color changes on the all tested composite after immersion with distilled water throughout the 4-week study. Figure 2 shows ∆*E* of each type of composite increased over the four weeks of the immersion period without following a similar fashion.

The discoloration effects of the beverages solution on the composites tested after each immersion period are also shown in Figure 2. As for coco-based drink and distilled water, in between weeks 1 and 4, no significant difference in color changes was observed on of all composites with GUF and CXO, showing clinically perceptible ∆*E* across the duration of study. For the coffee, there is significant difference in color changes of all composites (*p* < 0.05), except SX after 1 week and 4 week. Meanwhile, in the same evaluation period, Chinese tea showed significant difference in color changes of all composites except the CXO and GUF composite, demonstrating the lowest discoloration effect of CXO and GUF by this solution. NFL is the only composite tested with significant difference in color changes after immersion in milk tea at week 1 and week 4.

## 4. Discussion

In the present study, the color stability of five methacrylate-based composites was assessed with the effect of immersion in five different beverages by using the reflectance spectrophotometer based on the CIE *L*^*∗*^*a*^*∗*^*b*^*∗*^ color system. Here, we were trying to analyze the effect of composition of each composite on staining ability. Therefore, finishing and polishing of the samples was not included in the present study as different polishing systems might result in different surface roughness in different materials, which might subsequently affect the final stain uptake in the materials [12, 13]. There are two major thresholds used to evaluate color in dentistry which is perceptibility threshold (PT) and acceptability threshold (AT). These are two quality controls tools to evaluate the selection and clinical visual performance of dental materials. A recent study by Paravina et al. [11] had demonstrated that CIELAB PT and AT in dentistry was found to be at 1.2 and 2.7 color unit, respectively, with the 50 : 50% confidence level. Therefore, in this study, Δ*E* values greater than 2.7 color units are considered clinically unacceptable.

In the present study, some of the tested composite materials revealed significantly different color changes after 4 weeks of an immersion study. Whilst CXO and GUF demonstrate the least affected composite materials, SX had the highest discoloration effects after immersion in 4 types of commonly consumed local beverages. It is also in accordance with recent studies by Erdemir et al. [14] that different staining solutions demonstrated different effects of discoloration on the composite tested over time. Therefore, both null hypothesis of the study were rejected.

Color stability of a composite is closely related to the hydrophobic nature or hydrophilic nature of a composite, which is greatly influenced by the type of the resin matrix [13, 15]. Excessive water sorption of the material can cause formation of microcracks or interfacial gaps at the filler-matrix interface, as a result of hydrolytic degradation of the filler and filler-matrix debonding [6]. These microcracks act as the penetration pathway for stain with water molecules acting as the vehicle [16]. For instance, the presence of highly condensed hydrophobic polysiloxane backbone in CXO seems to have contributed to its high color stability. This finding was in accordance with the study conducted by Arocha, Mayoral [2]. However, in the present study, the color stability of RHC was higher than that of NFL although it is composed of both Bis-GMA and TEGDMA, while NFL is solely made up of Bis-GMA. The incorporation of the new innovative rice husk nanosilica might be contributing to the higher color stability in RHC, which is noteworthy for further study [8].

As expected, the higher content of TEGDMA in RHC (40 wt.%) has led to its lower color stability when compared to GUF, which is only composed of 5–10 wt.% of TEGDMA. In GUF, the presence of UDMA appeared to have rendered this composite more color stability than the other composites as UDMA is known to be less susceptible to water sorption due to its limited hydrophilic aliphatic chains [17]. However, being a microhybrid composite, the presence of UDMA in SX seems to be ineffective in making it more stain resistant than the other nanohybrid composites.

The importance of the filler particle size in affecting the color stability of a composite has been reflected in the case of SX. According to Fontes and Fernández [13], composite with small filler size might impart high color stability in the material. On the contrary, composites with large filler particles have higher tendency for water aging discoloration and are more susceptible to staining [12]. Comparing the filler size of the composites in the present study, CXO has the smallest filler size (2.3 nm), followed by NFL (10 nm), GUF (16–200 nm), RHC (261 nm), and SX (20–30 *µ*m). CXO with the smallest filler size was found to have the highest color stability, while SX being a microhybrid composite with the greatest filler size was found to have the lowest color stability. This finding was further supported with the results of study conducted by Moraes et al. [18] in which microhybrid composite was found to manifest the highest water sorption and highest color changes, as compared to nanofilled and nanohybrid composites. However, Gönülol and Yılmaz [19] reported in their study that composites with smaller fillers do not necessary exhibit low discoloration. The results of present study revealed that the color stability of a composite increased with the reduction in the filler size, except for NFL. Most probably due to the presence of Bis-GMA resin in NFL, its color stability was being surpassed by GUF and RHC despite having the smallest filler size second to CXO.

The filler loading of the composite has also been shown to influence the color stability of the material. Previous studies suggested that composite with high color stability comes along with high filler loading [3, 12]. Inorganic glass is followed by CXO (57 vol.%), NFL (54 vol.%), GUF (50 vol.%), and RHC (50 vol.%). Among the nanohybrid composites, the color stability of the composite increases as the filler loading increases, except for NFL. Despite the relatively higher filler loading in NFL, its color stability paled in comparison with GUF and fillers are unable to absorb water into the bulk of material; instead, it can only absorb water at the surface [20]. Comparing the filler loading of the composites in the present study, SX has the highest filler loading (65 vol.%), RHC probably due to the presence of Bis-GMA resin in the material.

Immersion period also played an integral part in affecting the color changes of the composites. Ertas et al. [21] have reported that 24 hours of staining *in vitro* is equivalent to about one month of staining in vivo. In the present study, samples were immersed in beverages for four weeks in order to simulate 2.5 years of clinical aging [6].

It is pertinent to emphasize that the exact correlation between in vitro and in vivo tests is hard to establish. Future studies need improvisations to simulate the staining ability of the composite in the oral cavity by considering the complexity of dietary colorants, thermal stress, pH variation, and dynamics of fluid flow in the oral cavity. The intermittent nature of exposure to solutions and the diluting effect of saliva in the oral cavity need to be considered before clinically transferring the results on staining effects of the composites.

## 5. Conclusion

Within the limitations, it can be concluded that the color stability of a composite is influenced by its composition (resin matrix), filler size, filler loading, the type of beverage consumed, and the duration of exposure to the beverage. The rice husk composite being an environmentally friendly option though not ideal may be considered for aesthetic restorations. Future research can be targeted to produce a hydrophobic composite inheriting the nanoceramic technology and incorporating the environmental-friendly and economical rice husk nanosilica particles.

## Figures and Tables

**Table 1:** Composition of methacrylate-based composites investigated in the study.

Composite	Resin	Filler	Manufacturer	Lot
G-aenial Universal Flo (GUF)	UDMA, Bis-MEPP, TEGDMA	Silicon dioxide (16 nm), strontium glass (200 nm), pigment	GC Dental Products Corporation, Japan	1503000787 1412000532
Solare-X (SX)	UDMA, dimethacrylate	Silica nanoparticles, fluoroaluminosilicate glass fillers, prepolymerised fillers (lanthanoid fluoride nanoparticles, silica nanoparticles, and fluoroaluminosilicate glass fillers)	GC Dental Products Corporation, Japan	1403272 1403262
Ceram.X One Universal (CXO)	Methacrylate-modified polysiloxane, dimethacrylate resin	Barium-aluminium-borosilicate glass (1–1.5 *µ*m), methacrylate-functionalized silicon dioxide nanofiller (10 nm), organically modified ceramic nanoparticles (2–3 nm)	Dentsply DeTrey GmbH, Germany	1503000787 1412000532
Neofil (NFL)	Bis-GMA	Barium borosilicate glass, silica-zirconia nanoparticle (0.01 microns)	Kerr Corporation, USA	5296721
Rice husk composite (RHC)	Bis-GMA, TEGDMA	Nanosilica obtained from rice husk, silanized with 6 wt.% *γ*-MPS	USM, Malaysia	—

UDMA: diurethane dimethacrylate; Bis-MEPP: bisphenol-A ethoxylate dimethacrylate; TEGDMA: triethylene glycol dimethacrylate; Bis-GMA: bisphenol-A glycidyl methacrylate; y-MPS: 3-(trimethoxysilyl)propyl methacrylate.

**Table 2:** Composition of staining solutions investigated in the study.

Staining solution	Ingredients	Manufacturer	Concentration
Coco-based drink (CHO)	Extract of malted barley, maltodextrin, cow milk, sugar, cocoa, palm oil, minerals, vitamin	Milo, Nestlé, Malaysia	33 g of Milo/200 ml boiling distilled water
Coffee (COF)	Sugar, glucose, syrup, hydrogenated palm kernel oil, instant coffee, skimmed milk powder 4% (cow milk), sodium caseinate (milk protein), salt	Nescafé Blend & Brew™, Nestlé, Malaysia	20 g of Nescafe/180 ml boiling distilled water
Chinese tea (CHI)	Pu-erh tea leaves	Pu-erh tea (M) SDN BHD, Malaysia	2 g of pu-erh tea/40 ml boiling distilled water
Milk tea (MT)	Nondairy creamer, sugar, instant tea, stevia leave extract	Teh tarik original, BOH, Malaysia	27 g of teh tarik/180 ml boiling distilled water
Distilled water	—	Hamilton Laboratory Glass Ltd, UK	As supplied

**Table 3:** Color changes of composites at the end of four weeks.

Composite	CHO	COF	CHI	MT	Control
GUF (A)	1.45 (0.76)^a,b^	4.44 (0.61)^a^	4.85 (0.34)^a^	1.66 (0.17)^a^	0.55 (0.33)^a^
SX (C)	2.83 (1.88)^b^	7.70 (1.16)^b^	22.89 (0.91)^c^	15.73 (3.58)^c^	1.06 (0.53)^a^
CXO (A)	0.80 (0.44)^a^	4.65 (0.73)^a^	3.29 (1.12)^a^	1.21 (0.19)^a^	0.56 (0.48)^a^
NFL (C)	3.78 (0.34)^c^	14.38 (0.72)^c^	20.43 (1.61)^c^	4.80 (0.47)^b^	0.50 (0.14)^a^
CX (B)	3.46 (1.08)^c^	12.96 (2.04)^c^	13.11 (3.19)^b^	2.91 (0.40)^a,b^	0.90 (1.26)^a^

^a–c^Mean values followed by different superscript letters and composites followed by different uppercase letters in the same column are statistically different according to Tukey's post hoc test (*p* ≥ 0.05).

## Data Availability

The color assessment data used to support the findings of this study are available from the corresponding author upon request.
